# Genetic signature related to heme-hemoglobin metabolism pathway in sepsis secondary to pneumonia

**DOI:** 10.1038/s41540-019-0105-4

**Published:** 2019-08-01

**Authors:** Giuseppe Gianini Figuerêido Leite, Brendon P. Scicluna, Tom van der Poll, Reinaldo Salomão

**Affiliations:** 1 Division of Infectious Diseases, Department of Medicine, Hospital São Paulo, Escola Paulista de Medicina, Universidade Federal de Sao Paulo, São Paulo, Brazil; 2 Center for Experimental and Molecular Medicine, Amsterdam University Medical Centers, location Academic Medical Center, University of Amsterdam, Amsterdam, The Netherlands; 3 Center of Infection and Immunity Amsterdam, Amsterdam University Medical Centers, location Academic Medical Center, University of Amsterdam, Amsterdam, The Netherlands

**Keywords:** Computational biology and bioinformatics, Genetic interaction, Immunology, Molecular medicine

## Abstract

Sepsis is defined as a life-threatening organ dysfunction caused by a dysregulated inflammatory response to pathogens. Bioinformatics and transcriptomics studies contribute to get a better understanding of the pathogenesis of sepsis. These studies revealed differentially expressed genes (DEGs) in sepsis involved in several pathways. Here we investigated the gene expression profiles of blood leukocytes using three microarray datasets of sepsis secondary to pneumonia, focusing on the heme/hemoglobin metabolism pathway. We demonstrate that the heme/hemoglobin metabolism pathway was found to be enriched in these three cohorts with four common genes (*ALAS2*, *AHSP*, *HBD*, and *CA1*). Several studies show that these four genes are involved in the cytoprotection of non-erythrocyte cells in response to different stress conditions. The upregulation of heme/hemoglobin metabolism in sepsis might be a protective response of white cells to the hostile environment present in septic patients (follow-up samples).

## Heme-hemoglobin metabolism pathway in sepsis secondary to pneumonia

Sepsis is defined as a life-threatening organ dysfunction caused by a dysregulated inflammatory response to pathogens. A study led by Professor Reinaldo Salomão (Universidade Federal de São Paulo) used three publicly microarray datasets of sepsis secondary to pneumonia and different bioinformatics tools to shed light on the importance of heme/hemoglobin metabolism in sepsis. Thus, four common genes were found upregulated in the three datasets (ALAS2, AHSP, HBD and CA1), notably, they were most significantly expressed in follow-up samples of patients who survived sepsis. This might be a cytoprotective response of white cells to the sepsis’ hostile environment. Future studies addressing the heme-hemoglobin metabolism pathway, focusing in these four genes, in a heterogeneous cohort of patients and collected on different days after hospital admission, would help to understand its modulation in sepsis and a possible role for protecting cells from stress conditions found in sepsis.

## Introduction

Sepsis is one of the most common causes of death in hospitalized patients and has an increasing burden as a result of population ageing and associated comorbidities.^1,2^ Sepsis has been defined as a life-threatening organ dysfunction caused by a dysregulated and uncontained host inflammatory response to pathogenic agents.^3^ Epidemiological studies have shown an increase in the global sepsis incidence, representing a severe health burden due to morbidity, mortality, and the high cost of septic and post-septic patients care.^4,5^

Sepsis results from complex interactions between the human host response and infecting microorganisms in which host mechanisms are involved in the pathophysiology of the syndrome and play a crucial role in their clinical manifestations.^6,7^ In recent years, transcriptomics profile and bioinformatics techniques have been used to provide a comprehensive understanding of the pathophysiology of sepsis.^8–10^ These studies revealed differentially expressed genes (DEGs) involved in cytokine signaling pathways, antigen presentation, the mitochondrial respiratory chain pathway and heme/hemoglobin metabolism pathway.

The role of heme as part of extracellular hemoglobin was described in sepsis, infections and in critically ill patients, mainly as a pro-inflammatory signaling molecule with binding specificity to Toll-like receptor 4 (TLR4).^11,12^ Besides the role of hemoglobin as the major oxygen carrier, several other functions have been characterized, including modulation of redox functions and interactions with gaseous transmitters such as nitric oxide and hydrogen sulfide.^13^ Thus, its presence has been shown in several other non-erythroid cells in stress conditions.^13,14^ Nevertheless, gene expression related to the heme/hemoglobin metabolism pathway and its relation with white cells is poorly characterized in sepsis. In fact, in patients with respiratory symptoms suspected of having community-acquired pneumonia (CAP), heme biosynthesis was among the main pathways corresponding to upregulated genes that were present in CAP-patients and not in non-CAP (without any other infection) patients.^15^

In the present study, to avoid heterogeneity of multiple primary sources of sepsis, we investigated the gene expression profiles of sepsis patients caused by CAP and/or hospital-acquired pneumonia (HAP), focusing in heme/hemoglobin metabolism in white cells.

## Results

### Initial screening using gene co-expression network (GCN)

As an initial screening of the data, a GCN was built using the S1 dataset, which is composed of 20 samples of peripheral blood mononuclear cells (PBMCs) from CAP patients. For analysis of DEGs, the S1 dataset has been divided into four groups, according to patients’ outcome (10 survivors and 10 non-survivors samples) and each sample collection day (D0 and D7) (Table 1 and Supplementary Material 1 pp. 1). Based on the DEGs list of the S1 dataset (Table 2), four GCN were constructed. In each network, modules were generated and analyzed. Modules analysis in GCN is used to group genes with similar expression patterns, generating clusters of genes in which they often share the same biological processes (BP).^16^

**Table 1:** Information about the datasets^a^

Access	Patients/controls	Samples	Platforms	References
GSE48080 (S1)	10 patients and 3 controls	PBMCs^b^	Agilent-014850	Severino, et al.^49^
E-MTAB-5273 (S2)	38 patients and 10 controls	Leukocytes	Illumina HumanHT-12 v4	Burnham, et al.^10^
GSE65682 (S3)	181 patients and 42 controls	Whole blood	Affymetrix U219 Array	Scicluna, et al.^9^

^a^More information on datasets can be seen in Supplementary Information 1

^b^PBMCs Peripheral blood mononuclear cells

**Table 2:** DEGs and molecular signature related to heme metabolism in S1 dataset

Group	D0S	D0NS	D7S	D7NS
N° DEGs	255	193	187	114
↑/↓	↑121 and ↓134	↑137 and ↓56	↑99 and ↓88	↑71 and ↓43
FDR	2.11E−07	NE^a^	9.96E−14	1.84E−03
Enrichment position	3rd of 25	NE^a^	1st of 12	3rd of 13
N° Genes	11	1	15	5
	*↑ AHSP*	*↑H1F0*	*↑AHSP*	*↑ ALAS2*
	*↑ ALAS2*		*↑ALAS2*	*↑ EPB42*
	*↑ CA1*		*↑CA1*	*↑ HBD*
	*↑ EPB42*		*↑EPB42*	*↑ SELENBP1*
	*↑ GYPA*		*↑ FAM46C*	*↑ SLC4A1*
	*↑ HBD*		*↑ GYPA*	
	*↑ RBM5*		*↑ HBD*	
	*↑ RHCE*		*↑ RAP1GAP*	
	*↑ SELENBP1*		*↑ RHCE*	
	*↑ SLC25A37*		*↑ SELENBP1*	
	*↑ SLC4A1*		*↑ SLC25A37*	
			*↑ SLC4A1*	
			*↑ SLC6A8*	
			*↑ SPTA1*	
			*↑ TNS1*	

D0S = Day 0 surviving group, D0NS = Day 0 Non-surviving group, D7S = Day 7 surviving group and D7NS = Day 7 Non-surviving group; ↑ Upregulated genes, and ↓ downregulated genes

^a^*NE* not enriched (the pathway was not found within the established parameters due to the small amount of genes related to it in this group)

**Fig. 1:**
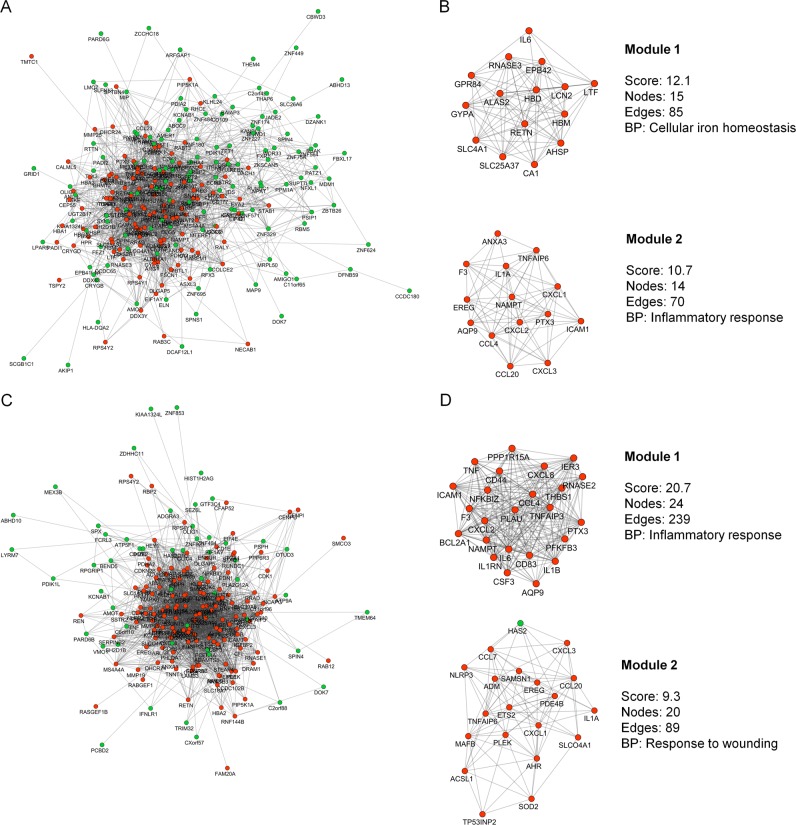
Representative co-expression networks of groups of patients on day 0 in S1 dataset. **a** GCN with DEGs of the D0S group. **b** Modules of the D0S group. **c** GCN with DEGs of the D0NS group. **d** Modules of the D0NS group. Red nodes represent the upregulated DEGs while green nodes represent the downregulated DEGs. The edge between them represents the probability of co-expression. BP biological process

In admission samples of surviving patients (D0S), the co-expression network is composed of 240 nodes and 1592 edges (Fig. 1a). After analysis with MCODE^17^ we found two modules, the first one being enriched for the BP related to “cellular iron homeostasis” and the other to “inflammatory response” (Fig. 1b). The GCN of admission samples of non-surviving patients (D0NS) is represented by 186 nodes and 1940 interactions between them (Fig. 1c). In this network two modules were found, one related to the “inflammatory response” and the other to “response to wounding” (Fig. 1d).

**Fig. 2:**
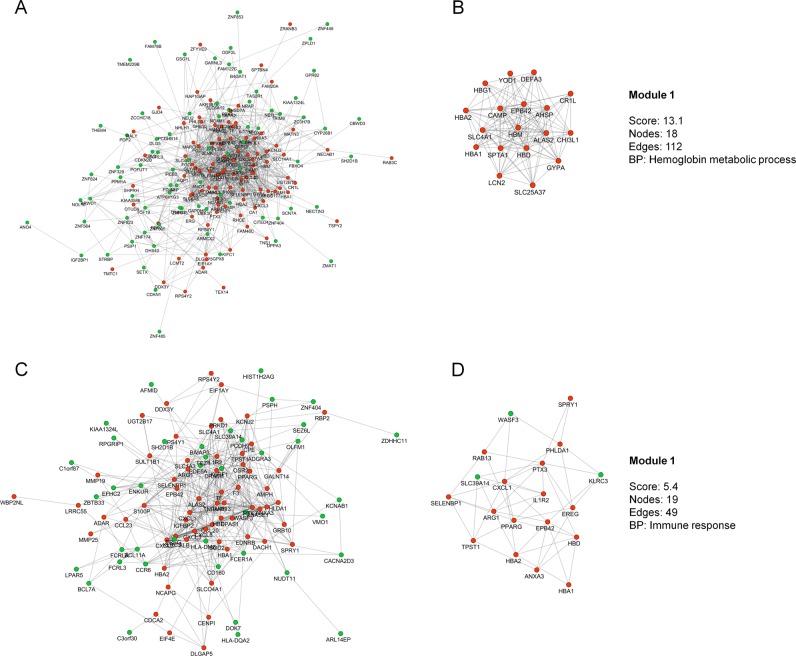
Representative co-expression networks of groups of patients on day 7 in S1 dataset. **a** GCN with DEGs of the D7S group. **b** Modules of the D7S group. **c** GCN with DEGs of the D7NS group. **d** Modules of the D7NS group. Red nodes represent the upregulated DEGs while green nodes represent the downregulated DEGs. The edge between them represents the probability of co-expression. BP biological process

In order to analyze sepsis progression, samples were collected seven days after diagnosis (D7S and D7NS). The D7S network consists of 174 nodes and 866 interactions (Fig. 2a). For this network, only one enriched module was found, related to “hemoglobin metabolic process” (Fig. 2b), which included genes common with those found in the D0S module, such as *ALAS2*, *AHSP* e *HBD*. Finally, the D7NS network presents 101 nodes and 415 interactions (Fig. 2c), the resulting module was enriched for the BP “immune response” (Fig. 2d).

### Molecular signature related to heme metabolism

The initial screening strengthened that heme/hemoglobin biosynthesis is modulated in sepsis and gave us insight in its possible effect on cytoprotection, since the modules related to “cellular iron homeostasis” and “hemoglobin metabolic process” are directly related to this biosynthesis pathway and were only found in surviving networks in S1 dataset. Therefore, subsequent analyses were performed using three datasets of sepsis secondary to pneumonia (S1, S2, and S3) (Supplementary Material 1, pp. 1–3). Full list of DEGs and molecular signatures for S1, S2, and S3 are shown in Supplementary Materials 2, 3, and 4, respectively.

In S1 dataset, 25 signatures were found in the D0S group, with the molecular signature related to heme metabolism being the 3rd most enriched, encompassing 11 genes. For the D0NS group, the heme-related signature was not found. For D7S, 12 signatures were found, the one related to heme being the most enriched with a total of 15 genes. For D7NS, 13 different signatures were found, the one related to heme metabolism was the 3rd more enriched with 5 genes (Table 2).

Thus, signatures of patient groups in the surviving groups (D0S and D7S) showed more genes related to the heme metabolism pathway than the groups of patients who did not survive (D0NS and D7NS).

The S2 dataset was divided into six groups according to day of sample collection (1, 3 and 5) and outcome (S and NS) (Supplementary Material 1, pp. 2). For the D1S and D1NS groups, 19 and 22 signatures were found, respectively, but the heme related signature was not found in any of them (Table 3). D3S group did present the signature related to heme metabolism, which was the 7th most enriched among 18 signatures found. For the D3NS group, the signature related to heme metabolism was not found among the 21 enriched signatures. An increase in the number of genes and in the signature position was observed in D5S group, in which the heme metabolism signature consisted of 10 genes and was the 2nd among 18 different signatures. For the D5NS group, 16 enriched signatures were found, but the one related to heme was not present.

**Table 3:** DEGs and molecular signature related to heme metabolism in S2 dataset

Group	D1S	D1NS	D3S	D3NS	D5S	D5NS
N° DEGs	224	274	159	268	173	184
↑/↓	↑132 and ↓92	↑144 and ↓130	↑101 and ↓58	↑158 and ↓110	↑109 and ↓64	↑111 and ↓73
FDR	NE^a^	NE^a^	5.10E−04	NE^a^	1.07E−07	NE^a^
Enrichment position	NE^a^	NE^a^	7th of 18	NE^a^	2nd de 18	NE^a^
N° Genes	3	3	6	4	10	3
	↑*ALAS2*	↑*ALAS2*	↑*AHSP*	↑*FAM46C*	↑*AHSP*	↑*FAM46C*
	↑*CA1*	↑*FAM46C*	↑*ALAS2*	↑*HBQ1*	↑*ALAS2*	↑*HBQ1*
	↑*E2F2*	↑*HBQ1*	↑*CA1*	↑*NFE2*	↑*CA1*	↑*SLC22A4*
			↑*FAM46C*	↑*RAP1GAP*	↑*FAM46C*	
			↑*HBD*		↑*GYPE*	
			↑*RAP1GAP*		↑*HBD*	
					↑*HBQ1*	
					↑*OSBP2*	
					↑*RAP1GAP*	
					↑*XK*	

D1S = Day 1 surviving group, D1NS = Day 1 Non-surviving group, D3S = Day 3 surviving group, D3NS = Day 3 Non-surviving group, D5S = Day 5 surviving group and D5NS = Day 5 Non-surviving group; ↑ Upregulated genes and *↓* downregulated genes

^a^*NE* not enriched (the pathway was not found within the established parameters due to the small amount of genes related to it in this group)

The S2 dataset follows a similar pattern to that observed in S1, with a higher number of genes and enrichment for the heme-related signature in the groups of patients who survived.

The S3 dataset was divided into two groups for analysis, one surviving group (SV) and one non-surviving group (NSV) (Supplementary Material 1 pp. 3). For the SV group, 40 enriched molecular signatures were found, the one related to heme metabolism being the 8th most enriched signature with 16 genes. For NSV group, 48 molecular signatures were found and the signature related to heme metabolism was the 10th most enriched with 19 genes (Table 4).

**Table 4:** DEGs and molecular signature related to heme metabolism in S3 dataset

Group	SV	NSV
N° DEGs	584	775
↑/↓	↑231 and ↓353	↑298 and ↓477
FDR	2.66E−08	5.33E−09
Enrichment position	8th of 40	10th of 48
N° Genes	16	19
	*↑ AHSP*	*↑ AHSP*
	*↑ ALAS2*	*↑ ALAS2*
	*↓ AQP3*	*↓ AQP3*
	*↑ BPGM*	*↑ BPGM*
	*↑ CA1*	*↑ CA1*
	*↑ GYPA*	*↑ GCLM*
	*↑ GYPB*	*↑ GYPA*
	*↑ HBD*	*↑ GYPB*
	*↑ ISCA1*	*↑ HBD*
	↑ LRP10	*↑ ISCA1*
	↑ MBOAT2	*↑ LMO2*
	↑ NUDT4	*↑ LRP10*
	↑ RHCE	*↑ MBOAT2*
	↑ SLC22A4	*↑ NUDT4*
	↑ SNCA	*↑ RHCE*
	↑ XK	*↑ SLC22A4*
		*↑ SNCA*
		*↑ TMCC2*
		*↑ XK*

*SV*surviving group, *NSV*non-surviving group; ↑ Upregulated genes and ↓ downregulated genes

### Identification of DEGs common among signatures

**Fig. 3:**
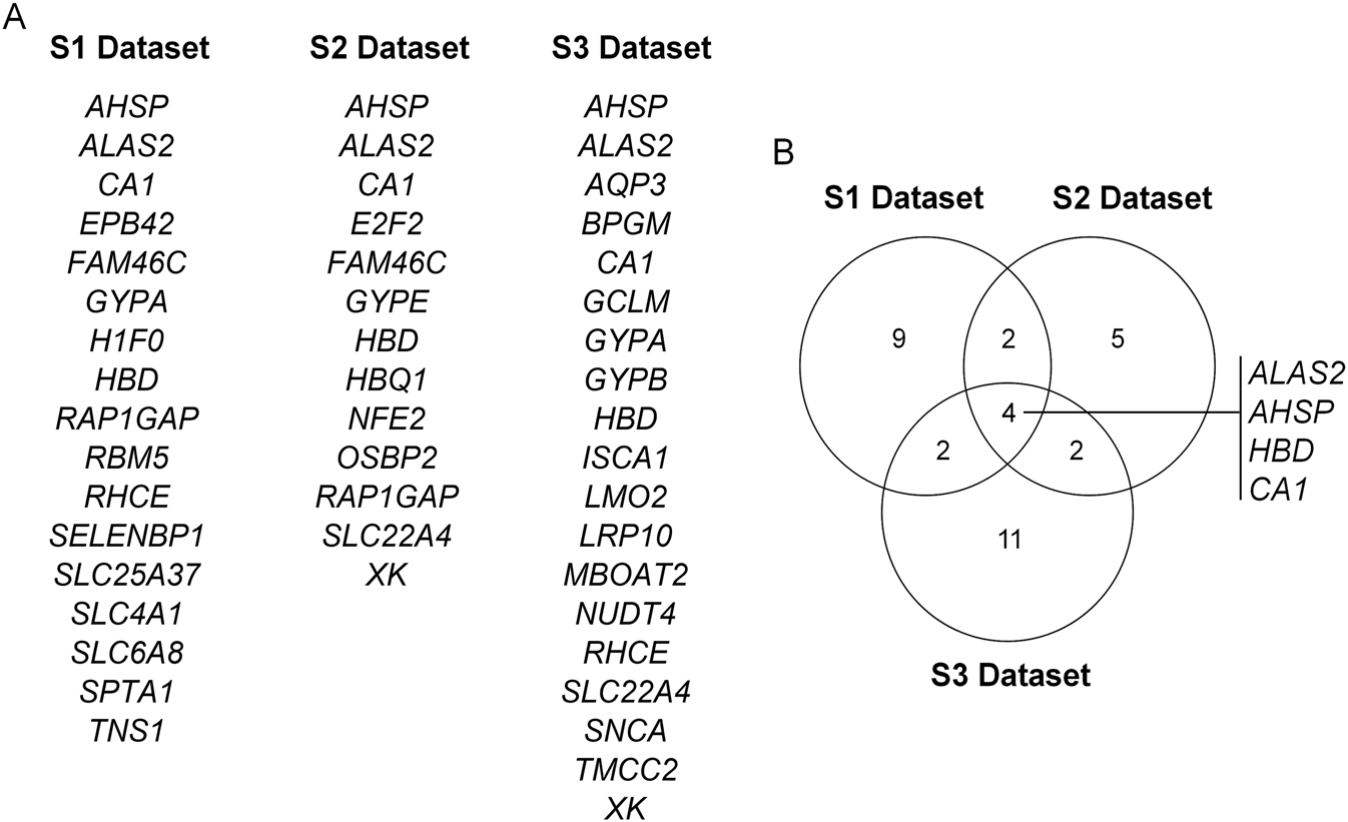
DEGs found in at least one of the groups for each datasets **a**; Venn diagram showing the common DEGs among the three datasets **b**

The DEGs that were found involved in the molecular signature related to heme metabolism in the different groups and in different datasets were overlapped using Venny 2.1 (Fig. 3a).

Through this analysis it was possible to find four common DEGs in the three datasets: *ALAS2* (5’-aminolevulinate synthase 2), *AHSP* (alpha hemoglobin stabilizing protein), *HBD* (hemoglobin subunit delta) and *CA1* (carbonic anhydrase 1) (Fig. 3b).

### Differential expression of the four common genes

**Fig. 4:**
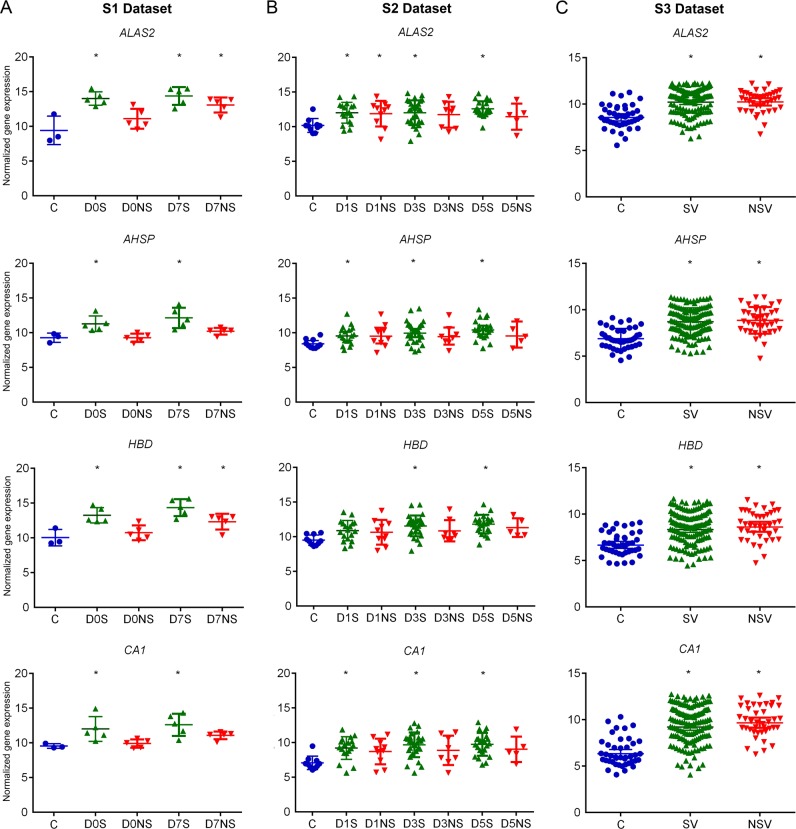
Gene expression variation for the four common DEGs. **a** S1 dataset, *p-value < 0.05 and Log_2_FC > 1.5; C control group, D0S day 0 surviving group, D0NS day 0 non-surviving group, D7S day 7 surviving group, and D7NS day 7 non-surviving group. **b** S2 dataset, *p-value, FDR < 0.05 and Log_2_FC > 1.5; C control group, D1S day 1 surviving group, D1NS day 1 non-surviving group, D3S day 3 surviving group, D3NS day 3 non-surviving group, D5S day 5 surviving group, and D5NS day 5 non-surviving group. **c** S3 dataset, * = *p*-value, FDR < 0.05 and Log_2_FC > 1.5; C control group, SV surviving group, and NSV non-surviving group (Supplementary Material 1 pp. 4–5)

The expression of the four DEGs was analyzed in each dataset. In S1 dataset, the *ALAS2 and HBD* showed a similar pattern of expression with an increase in expression in the D0S, D7S and D7NS groups. The *AHSP* and *CA1* genes exhibited increased expression only in surviving groups (Fig. 4). Overall, in S1 dataset the genes expression was more pronounced in surviving than in non-surviving groups.

The gene expression results for the four common DEGs in S2 were similar to those found in the S1 dataset. *ALAS2* expression value was increased in all surviving groups; and only in day one in the non-surviving group. The *AHSP* and *HBD* genes showed an increase in gene expression in D3S and D5S. The *CA1* gene was upregulated in all surviving groups. In S2, surviving septic patients showed a notable increase in the expression of these genes, substantially on days 3 and 5 (Fig. 4).

When analyzing the S3 dataset, we gained an increase in the number of patient samples, but we lost information regarding follow-up, since this dataset only contains samples collected in the first 24 h of admission to Intensive Care Units (ICUs). The S3 dataset exhibited a similar increase of expression for the four selected genes, all with a statistically significant FDR, regardless of the patientes’ outcome (Fig. 4).

When we directly analyzed surviving groups versus the non-surviving groups, in each dataset, this trend was not maintained. In the S1 dataset, the four common genes showed a statistically significant difference expression between surviving group versus the non-surviving group, except for ALAS2 in D7, more pronounced in D0S versus D0NS. No changes were observed between survivors versus non-survivors in S2 and S3 (Supplementary Material 1 pp. 6). Lack of differences in direct comparison might reflect the fact that these genes were in general upregulated in both groups, with increased magnitude in survivors relative to non-survivors, as has been previously reported by Xiao and coworkers in the transcriptome of complicated versus uncomplicated trauma patients.^18^

### Gene co-expression profile analysis of the four common genes (GCEPA)

**Fig. 5:**
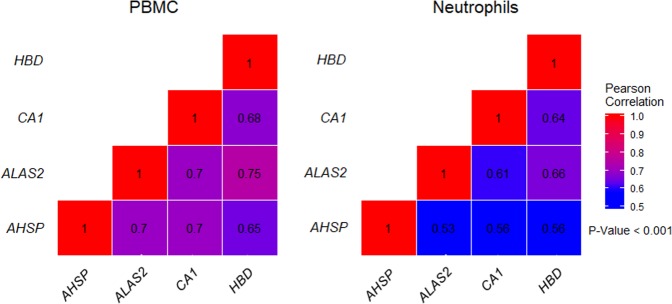
Heatmaps showing the gene co-expression ratio of the four common genes of the heme metabolism pathway in PBMC and Neutrophil samples (Supplementary material 1 pp. 7–8)

In order to support the gene expression modulation of the four common genes in white blood cells we performed a GCEPA using the Immuno-Navigator database which consists of a large collection of cell type-specific gene expression and co-expression data for cells of the immune system.^19^ The results obtained using human samples from this database showed a direct and statistically significant relationship (*p*-value < 0.001) between the expression of *ALAS2*, *AHSP*, *CA1,* and *HBD* in PBMC and neutrophil samples (Fig. 5). In general, PBMC had higher correlation values than the neutrophil samples, what can be explained, at least in part, by the number of samples (PBMC = 1120 and neutrophil = 228). The highest correlation value was observed between the *ALAS2* and *HBD* genes, both for PBMC (*r* = 0.75) and neutrophil samples (*r* = 0.66).

## Discussion

In our screening using GCN in the S1 dataset, we found the module enriched for the biological process of “cellular iron homeostasis” in D0S network, with the presence of genes directly related to the heme/hemoglobin metabolism, such as *ALAS2*, *AHSP*, *HBD*, and CA1. Similar biological process—“hemoglobin metabolic process”—was found in follow-up samples (D7S), containing three genes in common with those of the D0S module in addition to genes encoding for different globin chains of hemoglobin such as *HBA1*, *HBA2* e *HBG1*. No module related to the heme/hemoglobin was found in the network of the non-surviving groups.

Notably, a module similar to this was found in a network constructed from upregulated genes in human neonatal congenital cytomegalovirus infection, supporting modulation of these genes in infections.^20^

In attempt to provide evidence for modulation of heme/hemoglobin-related biosynthesis pathway in sepsis, and test the hypothesis that it might be related to outcomes, we analyzed three datasets of sepsis secondary to pneumonia (S1, S2, and S3) in the “*hallmark gene sets*” of MSigDB. Heme/hemoglobin-related biosynthesis signature was modulated in all datasets and contain the four genes (*ALAS2*, *AHSP*, *HBD*, and *CA1*) found in the modules in our screening with GCN.

*ALAS2* encodes a mitochondrial enzyme that regulates the initial step of heme biosynthesis.^21^ The *AHSP* is critical for the formation and stabilization of normal amount of hemoglobin.^22,23^ The *HBD* gene encodes the δ-globin, which together with the α-globin forms the tetramer HbA_2_^24^ and the *CA1* gene that encodes a protein that acts on the regulation of the affinity of hemoglobin for oxygen.^25^

Heme or hemoglobin-related genes are believed to be expressed in erythroid progenitors, but several studies have shown the expression of these genes in non-erythrocyte cells in response to different stress conditions, including murine macrophages and cervicovaginal epithelial cells from rabbits stimulated in vitro with LPS, IFN-γ, or hydrogen peroxide;^14,26^ granulation tissue induced by cellulose sponges^27^; PBMCs from patients with varying degrees of systemic inflammation, such as active systemic juvenile idiopathic arthritis^28^ and patients with cryopyrin-associated periodic syndromes.^29^ In addition, some studies have demonstrated the expression of genes related to the heme/hemoglobin in patients with a high degree of hypoxia^30^, as well as in murine alveolar cells submitted to hypoxia.^31^

Accordingly, inflammatory response, TNF-α, IFN-γ response, and hypoxia can be observed as altered molecular signatures—at different levels—in the three datasets studied (Supplementary material 1 pp. 9). The analyses of the *ALAS2*, *AHSP*, *HBD*, and *CA1* in a broad dataset of PBMCs and neutrophils, showing moderate to high correlations (Fig. 5), further support their gene expression modulation in white blood cells. In addition, it was demonstrated that the synthesis of proteins associated with hemoglobin is present in the process of monocyte-macrophage differentiation and decreases with the progress of differentiation.^32^

Our results with S1 and S2 datasets show that the heme/hemoglobin signature was related to outcomes, with increased expression in surviving groups. This association was not present for the S3 dataset, where no major differences were observed in relation to the patients’ outcomes. However, this dataset contains only admission samples and differences regarding patients’ outcomes in S1 and S2 datasets were more prominent in follow-up samples. Furthermore, datasets S1 and S2 were generated in PBMC and leukocytes, respectively, and dataset 3 was generated in whole blood. In fact, heme/hemoglobin signature was reported in this cohort of patients as part of the endotype with worst outcome.^9^

Heme is an essential molecule, involved in cellular physiology and metabolism. Nevertheless, in excess, free heme show cytotoxic effects and is characterized as a damage-associated molecular pattern (DAMP) activating TLR4 and inducing inflammation.^12^ In contrast, the upregulation of heme/hemoglobin pathway in white cells submitted to stress conditions might be related to its protective effects, and recent evidence support a role for heme-hemoglobin in this hostile environment.

Mitochondrial dysfunction has long been recognized to play an important role in organ dysfunction in septic patients.^33^ A study from our research group showed that genes encoding mitochondrial respiratory chain subunits, involved in oxidative phosphorylation, were more affected in non-surviving septic patients;^34^ interestingly, studies show that the expression of hemoglobin-related genes in non-erythrocyte cells play a role in mitochondrial function,^35^ and intracellular hemoglobin is preferentially located in the mitochondria, protecting it from hydrogen peroxide-induced cytotoxicity and mitochondrial DNA damage.^13^

We and others have shown that the production of reactive oxygen species and nitric oxide by monocytes and neutrophils is increased in septic patients and the persistence of this excessive production is related to poor outcomes.^36,37^ In the same sense, studies show that the hemoglobin expression in non-erythrocyte cells is related to an intrinsic mechanism of protection associated with the elimination of free radicals and detoxification to nitric oxide.^38^

This cytoprotective effect also occurs in response to hydrogen peroxide, which induces the increase in the expression of hemoglobin-related genes in hepatocytes^39^ and in rat mesangial cells^40^ as an antioxidative defense mechanism.

The inference of a protective effect for heme/hemoglobin signature is challenged by our previous report showing upregulation of this pathway in an endotype of worst outcome in septic patients.^9^ This discrepancy might be explained by the diverse approach to perform data analysis. In the present study we segregated patients in survivors and non-survivors for gene expression analyses, while in the endotype study patients were grouped throughout unsupervised cluster analysis, including in each group patients who survived and patients who did not survive. Anyway, these results add caution to interpret the presence of this signature in an individual patient.

We conclude that the heme and hemoglobin metabolism is modulated during sepsis, with emphasis in the four genes *ALAS2*, *AHSP*, *HBD,* and *CA1*, common to the three datasets. Their increased expression may be directly related to the white cells in response to adverse conditions present in septic patients such as infection, inflammation, hypoxia, and production of reactive oxygen species and oxidative stress. It might be a protective response to this hostile environment, an effect more evident in samples obtained in the course of the disease (follow-up samples).

## Methods

### Microarray datasets selection and data analysis

Sepsis datasets were selected according to the following criteria: age ≥ 18 years, sepsis secondary to pneumonia, presence of control group (healthy or individuals scheduled for elective procedures), and outcome information (patients who survived or did not survive after sepsis). Two datasets were deposited in GEO (Expression Gene Expression Omnibus) and one on ArrayExpress (Table 1).

These gene expression data were previously generated across from different platforms, thus, they were analyzed individually (Supplementary material 1, pp. 1–3). For the analysis of gene expression we used the R package LIMMA.^41^ In general, each dataset were processed as follows: the raw expression values had the background corrected, normalized and log2 transformed. Gene expression differences with *p*-value < 0.05 and with a |Log_2_ Fold-Change (FC)| > 1.5 between sepsis and controls were considered statistically significant in S1; FDR corrected with Benjamini–Hochberg procedure < 0.05 were used as additional cut-off for S2 and S3.

### Ethics approval and consent to participate

This study was not conduct on human biological specimens, all data were downloaded from public databases and therefore no authorization from the participants was required for this study. The study was approved by the ethics and research committee of the Universidade Federal de São Paulo (CEP: 0410.0087.04/2018; CAAE: 88055118.5.0000.5505) ensuring biological safety to conduct the study.

### Co-expression networks construction using GeneMANIA database

Biological networks in general are governed by graph theory. These graphs illustrate, in a systemic level, the complex data generated by technologies as transcriptomics.^42^ One of these graphs are the gene co-expression network (GCN), in this network, genes are represented as nodes, while the edges represent co-expression relation scored by Pearson correlation coefficient between two nodes.^16,43^

We build a GCN with the data of S1 as an initial screening of the data. Thus, networks were built with DEGs for the different days and outcomes present in this dataset (D0S, D0NS, D7S, and D7NS). For this purpose, only information derived from the co-expression category in the GeneMANIA database was used, the values of the interactions were maintained as default.^43^ These data have been downloaded, imported and viewed in Cytoscape 3.6.1.

### Network module detection

To identify molecular complexes, the Cytoscape MCODE^17^ plug-in was used with the following cut-off parameters: degree cutoff ≥ 15 and k-core > 4.0. Thereafter, the identified complexes were used for functional enrichment analysis using BinGO.^44^ The hypergeometric test was utilized for GO enrichment analyses with significance defined by Benjamini and Hochberg adjusted *p*-value < 0.05.^45^

### Molecular signature analysis related to heme metabolism

The analysis of the molecular signature relating to the heme metabolism to DEGS was performed using “Gene sets hallmark” from Molecular Signatures Database v 6.2 (MSigDB). This dataset represents well defined biological states or processes derived from the aggregation of many gene sets.^46^ The signatures were considered enriched when FDR <0.05.

The signature-related genes that were found for the different analyzes were overlaid using the Venny 2.1 software (http://bioinfogp.cnb.csic.es/tools/venny/), in order to find the common genes.

### Gene co-expression profile analysis of the common genes

The Gene co-expression Profile Analysis (GCEPA) was performed using the Immuno-Navigator database.^19^ The analysis was conducted as previously described.^47^ The Jetset database^48^ was employed to select more reliable probes from the Affymetrix HG-U133 Plus 2.0 chip for the common genes found. Only expression data from PBMC samples (*n* = 1120) and neutrophils (*n* = 228) were selected. Pearson’s two-tailed pairwise correlation was used to compare the co-expression relationship between the genes; a *P*-value < 0.01 was used as cut-off.

### Data presentation

The graphics were generated using GraphPad Prism 6.0, as well as ggplot2 package present in software R.

## Supplementary information

Supplementary Material 1

Supplementary Material 2

Supplementary Material 3

Supplementary Material 4

## Data Availability

The three sepsis transcriptomic datasets used in this study are freely available on the GEO data portal under the access GSE48080 and GSE65682; as well as on ArrayExpress under the access E-MTAB-5273. Cell type-specific gene expression and co-expression data for cells of the immune system are freely available on the Immuno-Navigator database (https://t.co/gzCjuYQYUf). The software and tools used in this study are publicly available (except GraphPad Prism 6.0). Custom codes used in the study are available upon request.

## Supplementary information

**Supplementary information** accompanies the paper on the *npj Systems Biology and Applications* website (10.1038/s41540-019-0105-4).
