# Supplementary material for: Genetic signature related to heme-hemoglobin metabolism pathway in sepsis secondary to pneumonia
Source: NPJ Syst Biol Appl. 2019 Aug 1;5:26. doi: 10.1038/s41540-019-0105-4 (PMC6672010; doi:10.1038/s41540-019-0105-4)
Supplement: Supplementary file 1 — Supplementary Material 1 [file 41540_2019_105_MOESM1_ESM.pdf]

1.1 Additional information about datasets and analyzes

1.1.1 Additional information about S1 dataset

The S1 dataset consists of 20 samples from adult patients admitted to Brazilian ICUs with sepsis due to community acquired pneumonia and 3 samples from healthy individuals (controls). PBMC samples were obtained using Ficoll gradient (Ficoll-Paque PLUS, GE Healthcare Life Sciences). The RNA was isolated using the Illustra RNAspin Mini kit (GE Healthcare Life Sciences) and then microarray analysis was performed using Agilent Whole Human Genome microarray platform 4×44K arrays and deposited in GEO under the accession number GSE48080.

Data were downloaded from GEO and were divided according to patients' outcome and each sample collection day (**Supplementary Table 1**). This data set is formed of patients whose samples were collected after admission to the ICU, within 48 hours of the first sign of organ dysfunction or septic shock, and classified in survivors (D0S) and not survivors (D0NS). In order to analyze the progression of sepsis, this dataset contains samples of the same patients obtained seven days after the first sample (D7S and D7NS). Healthy volunteers were used as controls.

Supplementary Table 1. Groups of the dataset GSE48080 (S1)

	**Nº of samples (D0)**	**Nº of samples (D7)**	**Average age**	**Ratio M/F***
**Surviving group (S)**	5	5	51	5/0
**Non-surviving group (NS)**	5	5	74	5/0
**Control group (C)**	3	0	59	2/1

*M = Male and F = Female

Samples from the S1 dataset were analyzed as follows: D0S x Control group, D0NS x Control group, D7S x Control group and D7NS x Control group. We also analyzed surviving groups versus non-surviving groups (D0S x D0NS and D7S x D7NS).

1.1.2 Additional information about S2 dataset

The S2 dataset originally consists of 227 samples from adult patients admitted to United Kingdom ICUs with sepsis due to community acquired pneumonia or faecal peritonitis and 10 samples of patients scheduled for heart surgery (controls). Leukocyte samples were obtained using the LeukoLOCK filters (Life Technologies, Carlsbad, CA) and subsequently the microarray analysis was performed using the Illumina HumanHT-12 v4 Expression BeadChips platform and deposited on the ArrayExpress under accession number E-MTAB-5273.

Data were downloaded from ArrayExpress and samples of septic patients secondary to pneumonia were selected. The samples were divided according to patients' outcome and each sample collection day (**Supplementary Table 2**). Thus, this dataset consists of patients whose samples were collected within 24 hours ICU admission and who survived (D1S) and did not survive (D1NS). This dataset contains patients' samples three and five days after ICU admission, who survived (D3S e D5S) and did not survive (D3NS e D5NS), as well as controls. One sample (CAP0205) was shown to be an outlier and was removed before proceeding to downstream analysis.

Supplementary Table 2. Groups of the dataset E-MTAB-5273 – S2

	Nº of samples (D1)	Nº of samples (D3)	Nº of samples (D5)	Average age	Ratio M/F*
**Surviving group (S)**	18	28	22	65	33/35
**Non-surviving group (NS)**	10	8	5	63	13/10
**Control group (C)**	10	0	0	67	8/7

*M = Male and F = Female

Samples from the S2 dataset were analyzed as follows: D1S x Control group, D3S x Control group, D5S x Control group, D1NS x Control group, D3NS x Control group and D5NS x Control group. We also analyzed surviving groups versus non-surviving groups (D1S x D1NS, D3S x D3NS and D5S x D5NS).

1.1.3 Additional information about S3 dataset

The S3 dataset originally consists of 760 samples from adult patients admitted to Netherlands ICUs with sepsis due to community acquired pneumonia, hospital-acquired pneumonia, hospitalized patients without infection, and 42 samples from healthy subjects (controls). Whole blood samples were collected using the PAXgene tubes (Becton-Dickinson, Breda, the Netherlands, RNA was isolated using the PAXgene blood mRNA kit (Qiagen, Venlo, the Netherlands) and then microarray analysis was performed using the Affymetrix Human Genome U219 array platform and deposited in GEO under the accession number GSE65682.

Data were downloaded from GEO and we selected only the samples of septic patients secondary to pneumonia. The samples were divided according to patients' outcome (**Supplementary Table 3**). Thus, this dataset consists of patients who were collected within 24 hours ICU admission and who survived (SV) and did not survive (NSV), as well as controls.

Supplementary Table 3. Groups of the dataset GSE65682 – S3

	**Nº of samples**	**Average age**	**Ratio M/F***
**Surviving group (SV)**	141	60	88/53
**Non-surviving group (NSV)**	40	64	22/18
**Control group (C)**	42	46	24/18

*M = Male and F = Female

Samples from the S3 dataset were analyzed as follows: SV x Control group and NSV x Control group. We also analyzed surviving groups versus non-surviving groups (SV x NSV).

1.2 Additional results about the differential expression of the four common genes

1.2.1 Differential expression of the four common genes comparing septic groups and controls

Supplementary Table 4. Gene expression variation for the 4 common DEGs S1 dataset

ALAS2			*AHSP*		
**Comparation**	**Log_2FC**	**P-value**	**Comparation**	**Log_2FC**	**P-value**
D0S-Control	4.58	3.82E-05 *	D0S-Control	2.00	5.71E-03 *
D0NS-Control	1.67	7.42E-02	D0NS-Control	-0.01	9.84E-01
D7S-Control	4.94	1.47E-05 *	D7S-Control	2.85	2.54E-04 *
D7NS-Control	3.64	4.95E-04 *	D7NS-Control	0.93	1.65E-01
HBD			*CA1*		
**Comparation**	**Log_2FC**	**P-value**	**Comparation**	**Log_2FC**	**P-value**
D0S-Control	3.23	3.53E-04 *	D0S-Control	2.44	6.04E-03 *
D0NS-Control	0.70	3.66E-01	D0NS-Control	0.34	6.72E-01
D7S-Control	4.31	1.16E-05 *	D7S-Control	3.05	9.97E-04 *
D7NS-Control	2.29	6.54E-03 *	D7NS-Control	1.52	7.20E-02

* = P-value < 0.05 and $Log_2FC > 1.5$; D0S = Day 0 surviving group, D0NS = Day 0 non-surviving group, D7S = Day 7 surviving group and D7NS = Day 7 non-surviving group.

Supplementary Table 5. Gene expression variation for the 4 common DEGs S2 dataset

ALAS2				*AHSP*			
**Comparation**	**Log_2FC**	**P-Value**	**FDR**	**Comparation**	**Log_2FC**	**P-Value**	**FDR**
D1S-Control	1.82	2.73E-03	1.20E-02 *	D1S-Control	1.20	3.55E-02	9.15E-02 *
D1NS-Control	1.69	1.36E-02	4.48E-02 *	D1NS-Control	1.22	6.21E-02	1.40E-01
D3S-Control	1.83	1.22E-03	6.76E-03 *	D3S-Control	1.63	2.51E-03	1.21E-02 *
D3NS-Control	1.54	3.32E-02	8.56E-02	D3NS-Control	1.12	1.04E-01	1.98E-01
D5S-Control	2.38	5.63E-05	5.87E-04 *	D5S-Control	2.07	2.26E-04	1.82E-03 *
D5NS-Control	1.24	1.36E-01	3.09E-01	D5NS-Control	1.22	1.26E-01	2.95E-11
HBD				*CA1*			
**Comparation**	**Log_2FC**	**P-Value**	**FDR**	**Comparation**	**Log_2FC**	**P-Value**	**FDR**
D1S-Control	1.36	2.54E-02	7.09E-02	D1S-Control	2.12	2.44E-03	1.10E-02 *
D1NS-Control	1.11	1.10E-01	2.11E-01	D1NS-Control	1.62	4.15E-02	1.04E-01
D3S-Control	2.03	3.91E-04	2.69E-03 *	D3S-Control	2.59	8.44E-05	7.56E-04 *
D3NS-Control	1.33	6.96E-02	1.46E-01	D3NS-Control	1.80	3.25E-02	8.44E-02
D5S-Control	2.29	1.22E-04	1.10E-03 *	D5S-Control	2.65	1.00E-04	9.42E-04 *
D5NS-Control	1.79	3.55E-02	1.32E-01	D5NS-Control	1.95	4.47E-02	1.54E-01

* = P-value, FDR < 0.05 and $Log_2FC > 1.5$; D1S = Day 1 surviving group, D1NS = Day 1 non-surviving group, D3S = Day 3 surviving group, D3NS = Day 3 non-surviving group, D5S = Day 5 surviving group and D5NS = Day 5 non-surviving group.

Supplementary Table 6. Gene expression variation for the 4 common DEGs S3 dataset

ALAS2				*AHSP*			
**Comparation**	**Log_2FC**	**P-Value**	**FDR**	**Comparation**	**Log_2FC**	**P-Value**	**FDR**
SV-Control	1.64	1.30E-11	1.05E-10 *	SV-Control	1.84	1.70E-12	1.50E-11*
NSV-Control	1.65	3.72E-08	2.57E-07 *	NSV-Control	1.98	8.39E-10	7.21E-09 *
HBD				*CA1*			
**Comparation**	**Log_2FC**	**P-Value**	**FDR**	**Comparation**	**Log_2FC**	**P-Value**	**FDR**
SV-Control	1.74	3.15E-09	2.00E-08 *	SV-Control	2.94	3.50E-17	4.62E-16 *
NSV-Control	1.98	6.16E-08	4.14E-07 *	NSV-Control	3.31	1.96E-14	2.85E-13 *

* = p-value, FDR < 0.05 and $Log_2FC > 1.5$; SV = Surviving group and NSV = Non-surviving group.

1.2.2 Additional results comparing surviving groups versus non-surviving groups

Supplementary Table 7. Surviving groups versus non-surviving groups analyses in S1 dataset

ALAS2			*AHSP*		
**Comparation**	**Log_2FC**	**P-value**	**Comparation**	**Log_2FC**	**P-value**
D0S-D0NS	2.91	1.00E-03 *	D0S-D0NS	2.01	1.00E-03 *
D7S-D7NS	1.3	0.10	D7S-D7NS	1.91	2.00E-03 *
HBD			*CA1*		
**Comparation**	**Log_2FC**	**P-value**	**Comparation**	**Log_2FC**	**P-value**
D0S-D0NS	2.52	9.00E-04 *	D0S-D0NS	2.1	6.00E-03 *
D7S-D7NS	2.01	5.00E-03 *	D7S-D7NS	1.53	3.00E-02 *

* = P-value < 0.05 and $Log_2FC > 1.5$; D0S = Day 0 surviving group, D0NS = Day 0 non-surviving group, D7S = Day 7 surviving group and D7NS = Day 7 non-surviving group.

Supplementary Table 8. Surviving groups versus non-surviving groups analyses in S2 dataset

ALAS2				*AHSP*			
**Comparation**	**Log_2FC**	**P-Value**	**FDR**	**Comparation**	**Log_2FC**	**P-Value**	**FDR**
D1S-D1NS	0.12	0.834	0.997	D1S-D1NS	-0.01	0.983	0.999
D3S-D3NS	0.28	0.634	0.886	D3S-D3NS	0.5	0.383	0.758
D5S-D5NS	1.13	0.130	0.999	D5S-D5NS	0.84	0.237	0.999
HBD				*CA1*			
**Comparation**	**Log_2FC**	**P-Value**	**FDR**	**Comparation**	**Log_2FC**	**P-Value**	**FDR**
D1S-D1NS	0.25	0.678	0.995	D1S-D1NS	0.5	0.460	0.994
D3S-D3NS	0.69	0.257	0.665	D3S-D3NS	0.79	0.260	0.669
D5S-D5NS	0.49	0.513	0.999	D5S-D5NS	0.7	0.419	0.999

D1S = Day 1 surviving group, D1NS = Day 1 non-surviving group, D3S = Day 3 surviving group, D3NS = Day 3 non-surviving group, D5S = Day 5 surviving group and D5NS = Day 5 non-surviving group.

Supplementary Table 9. Surviving groups versus non-surviving groups analyses in S3 dataset

ALAS2				*AHSP*			
**Comparation**	**Log_2FC**	**P-Value**	**FDR**	**Comparation**	**Log_2FC**	**P-Value**	**FDR**
SV-NSV	-0.006	0.978	0.997	SV-NSV	-0.14	0.568	0.944
HBD				*CA1*			
**Comparation**	**Log_2FC**	**P-Value**	**FDR**	**Comparation**	**Log_2FC**	**P-Value**	**FDR**
SV-NSV	-0.24	0.393	0.899	SV-NSV	-0.36	0.264	0.841

SV = Surviving group and NSV = Non-surviving group.

1.4 Additional results to the Pearson's correlation coefficient analyses

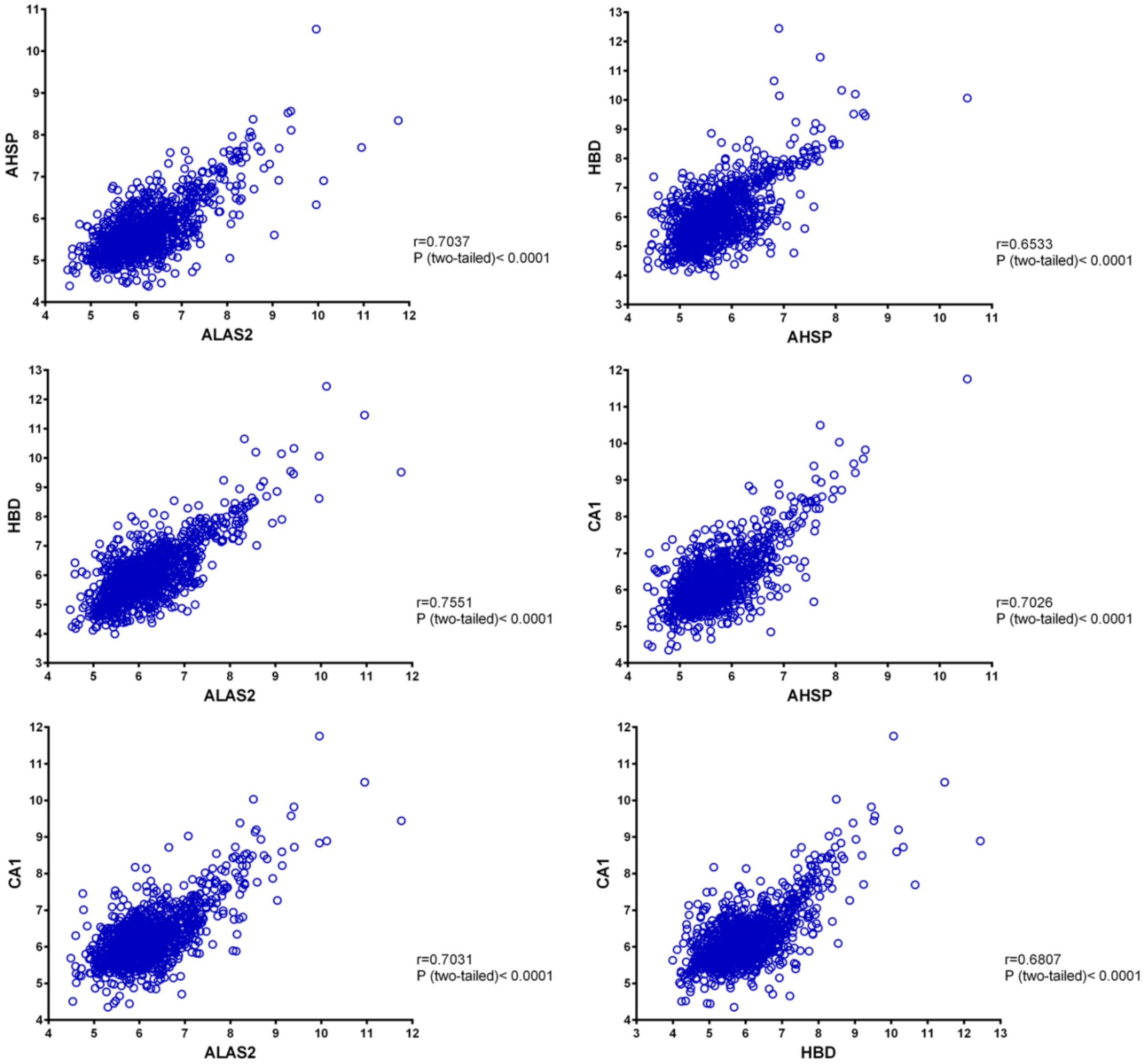

Supplementary Figure 1. Pearson correlation between the expression of four common genes in S1, S2 and S3 in PBMC samples from Immuno-Navigator database.

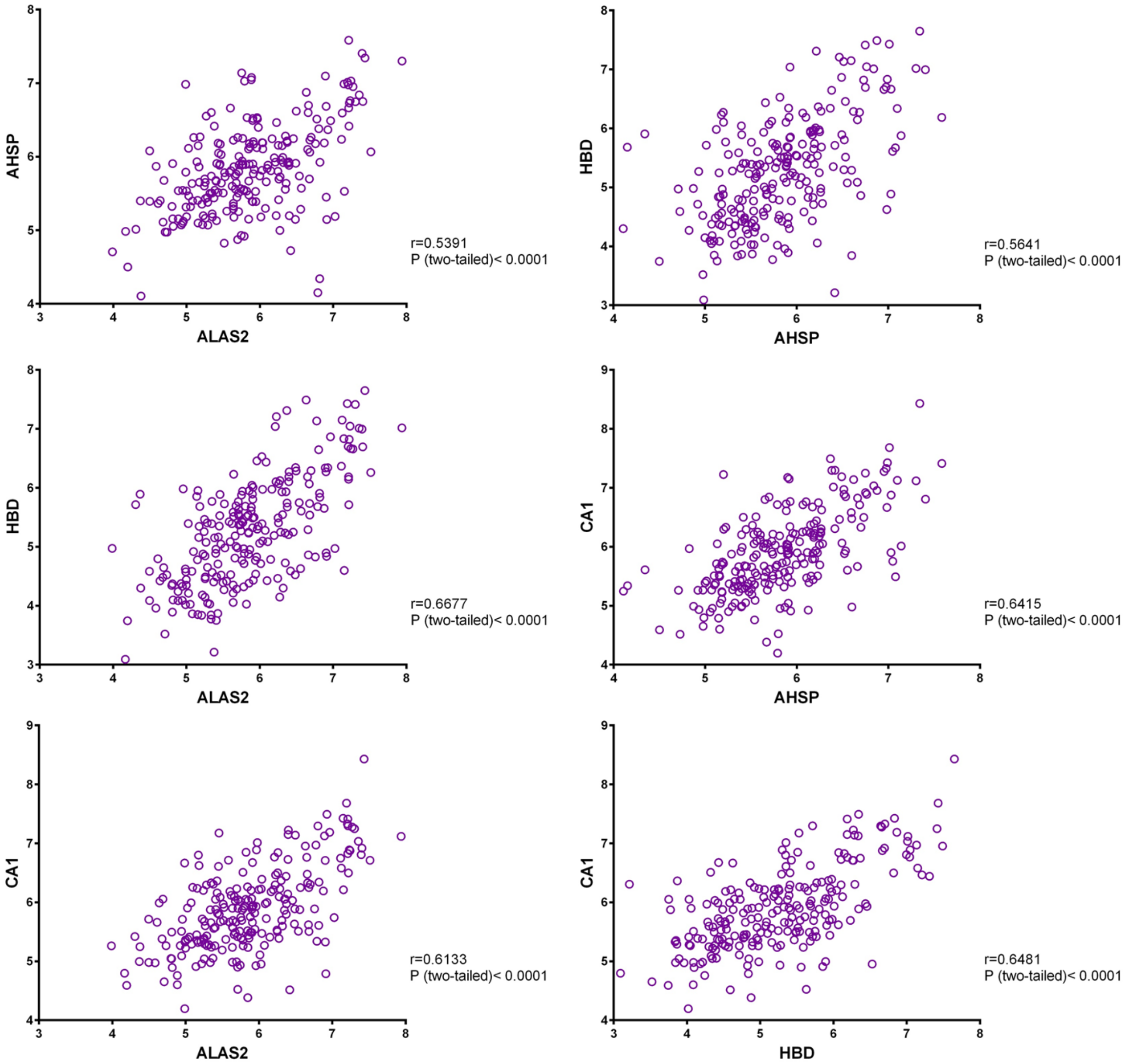

Supplementary Figure 2. Pearson correlation between the expression of four common genes in S1, S2 and S3 in neutrophil samples from Immuno-Navigator database.

1.5 Additional results to the HALLMARK of MSigDB

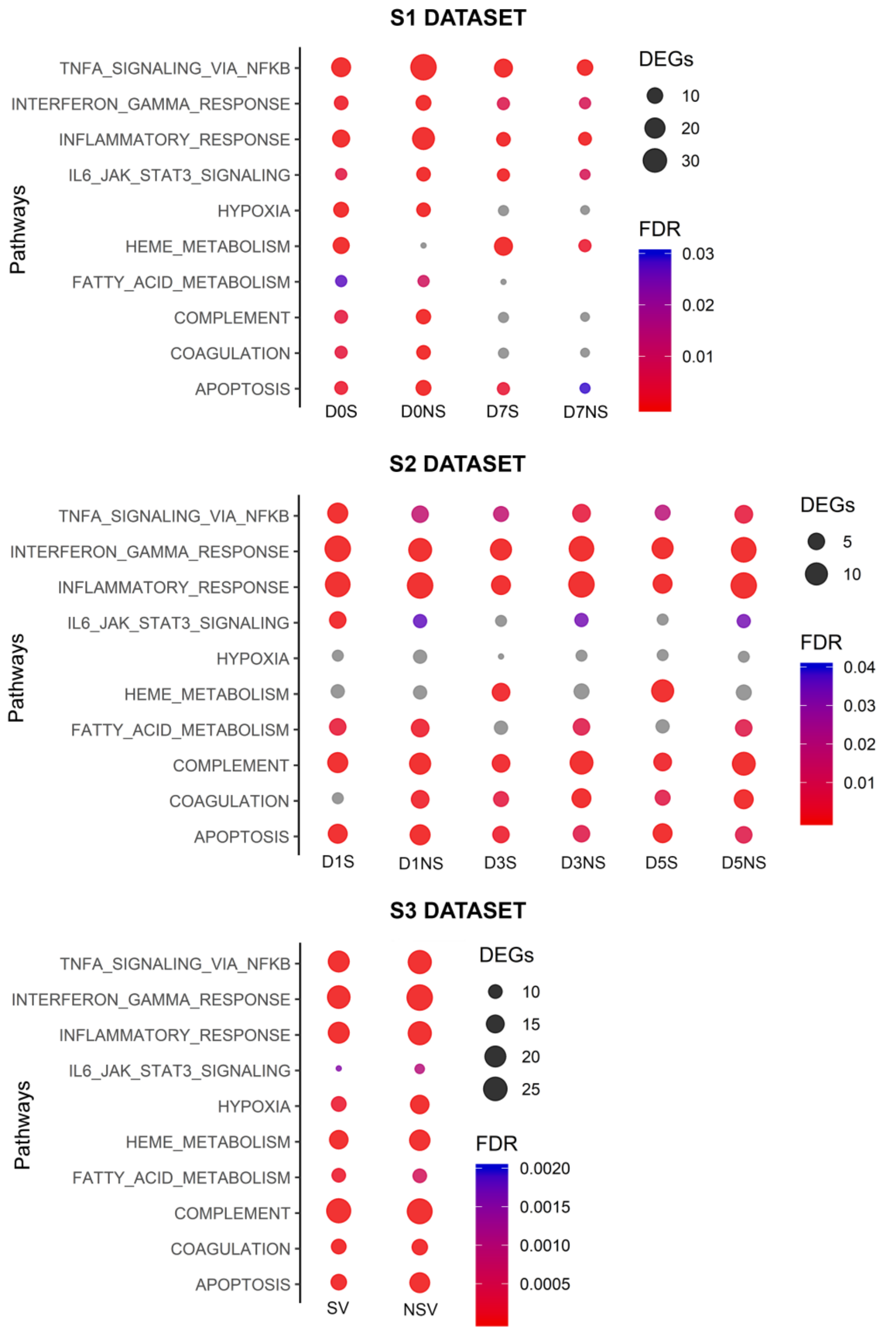

Supplementary Figure 3. Dotplot for enriched HALLMARK in the three datasets (S1, S2 and S3).
